# The “Muscles of the Psyche”: From Body Literacy to Emotional Literacy

**DOI:** 10.3389/fpsyg.2020.548964

**Published:** 2021-01-20

**Authors:** Maya Vulcan

**Affiliations:** Graduate School of Creative Art Therapies, Faculty of Humanities & Social Sciences, Kibbutzim College of Education, Tel Aviv, Israel

**Keywords:** autism, body, primary relations, somatic inter-subjectivity, the lived-experience of therapists, qualitative research

## Abstract

Autism spectrum disorder (ASD) is a neuro-developmental condition, which requires a multi-disciplinary matrix of treatments, including functional, educational, and emotional interventions. The latter mode of treatment entails particular difficulties, inasmuch as the core deficits of this condition seem to challenge the very premises of traditional psychotherapy. Reciprocity, verbal, and symbolic expression and inter-subjective dynamics are often difficult to attain with clients diagnosed with ASD, and emotional treatment thus often turns out to be a frustrating process, which may well elicit questions as to the efficacy of psychotherapeutic emotional interventions. These core challenges, described in the literature, become particularly acute in view of the increasing number of clients diagnosed on the autistic spectrum in recent years, and the growing need for qualified therapists who have trained for working specifically with this condition. It seems, therefore, that it is high time for systematic research into the lived experience of therapists working with these clients in order to attain a better clinical and theoretical understanding of the condition itself and broaden the range of effective interventions. This study, informed by a phenomenological-hermeneutic approach which guided both the collection of data and its subsequent analysis, aims to address these issues by exploring the particular challenges faced by therapists in this field, the questions that come up in the process, modes of personal and professional coping, and the insights elicited by the therapeutic encounter. The research consisted of in-depth interviews with 28 practicing therapists from a broad range of clinical orientations, including dance/movement, arts, music, and drama therapists, clinical psychologists, and clinical social workers. The essential themes that emerged from the participants’ responses and the analysis of the findings lend support to theoretical and developmental approaches, which focus on the primacy and the foundational role of the concrete body in inter-subjective relationships and in the therapeutic process, and indicate the potential efficacy of somatic and kinetic interventions. The clinical implications of this study are thus highly relevant to the training and support of therapists working with ASD, who should be encouraged to develop greater receptivity to non-verbal modes of interaction in the therapeutic process.

“When I die and go to heaven, if I'm asked, ‘what have you given to this world?’, I'd say, ‘look here guys, I went all the way and got to the most fundamental roots of what it means to be a person’.” (Saar, psychologist, participant in the study)

## Introduction

Autism Spectrum Disorder (ASD) is a neuro-developmental condition, which is diagnosed as a spectrum of “persistent deficits in social communication and social interaction across multiple contexts” and “restricted, repetitive patterns of behavior, interests, or activities” (American Psychiatric Association, 2013, p. 53). The origin and precise nature of ASD have been a matter of controversy and debate for the past 70 years, and the theoretical diversity regarding the etiology and definitions of ASD is echoed and reflected in debates about the most appropriate modalities of treatment (Drucker, 2009; Verhoeff, 2013). Current debates extend to the very fundamental distinction between a neuro-diversity perspective that seeks to avoid an ableistapproach, and a more traditional perspective, which considers ASD as a condition or a spectrum disorder (Jaarsma and Welin, 2012; Kenny et al., 2016; Gyawali and Patra, 2019). Be that as it may, working with children diagnosed with ASD is a two-pronged endeavor. The “functional” modes of treatment are designed to improve the child’s ability to function in the external world, to acquire skills, and to perform developmental tasks, e.g., speech therapy, occupational therapy, and the educational framework.

Most prominent among the various treatment modalities are those that offer early intensive interventions, either developmental or behavioral. To name just a few examples:

1. ABA – Applied Behavior Analysis: a method of teaching skills and behaviors to people with autism by breaking tasks down into very small, sequential steps. ABA relies on the idea that behaviors are spurred by a person’s environment (Weitlauf et al., 2014).
2. DIR – A developmental approach developed by Stanley Greenspan. DIR – Developmental, Individual Differences, Relationship Based (DIR)/Floor-time model for working with children with special needs (Greenspan and Wieder, 2006).
3. RIT – Reciprocal Imitation Training (Ingersoll and Schreibman, 2006).
4. ESDM – Early Start Denver Model (Dawson et al., 2010).
5. Specialized occupational therapy and speech therapy, and educational programs such as TEACCH – Treatment and Education of Autistic and Communications-Handicapped Children Program – A popular intervention program in the United States based on “Structured Teaching.” It is cognitively based on the relative strength of individuals with autism in visual information processing (Ruberman, 2002; Mesibov and Shea, 2010).

Alongside these “functional” modes of treatment, it is broadly recognized that the emotional aspects of the autistic condition call for psychotherapy, and various modes of “emotional” therapy focus on the inner reality of the child, the development of the self, the child’s attempts to express him/herself, his/her anxieties, inhibitions, defenses, and the way in which she/he perceives and experiences the world. Most of the research on ASD relates to psychotherapies, such as personal construct theories, family-focused therapy, and cognitive behavioral therapy (CBT; Hare, 1997; Cardaciotto and Herbert, 2004; Wood et al., 2015; Murphy et al., 2017; Storch et al., 2020). CBT takes center stage in research with ASD and numerous randomized controlled trials (RCTs) have shown that it is effective for treating anxiety disorders in adults, adolescents, and in children diagnosed with ASD (Sukhodolsky et al., 2013; Hesselmark et al., 2014; Vasa et al., 2014; Spain et al., 2015; Wood et al., 2015). On the whole, current therapeutic interventions for ASD are generally based on theoretical formulations supported by empirical research and developments in genetics, neuropsychiatry, neuropsychology, and educational and behavioral techniques, with preference for modes of therapy which can be evaluated through evidence-based research (Mesibov and Shea, 2011; Thompson, 2013). This, in turn, means that both the relevance and the efficacy of emotional therapy – informed by psychoanalytic and psychodynamic approaches – are sometimes questioned by practitioners and researchers alike, inasmuch as these traditional approaches do not lend themselves easily to empirical evaluation (Koeing and Levine, 2011). These tendencies notwithstanding, it is still broadly recognized that the autistic condition entails emotional challenges that must be addressed in therapy, issues of emotionality and identification, deficits in theory-of-mind and empathizing (Baron-Cohen et al., 1985), and the core problems that interfere with the maturation of intersubjectivity (Haag et al., 2005; Mitrani, 2010). In view of the fact that precisely these emotional capacities that constitute the building blocks of traditional emotional therapy are often impaired in ASD, it is inevitable that the search for relevant tools should be plagued by the question of “how are child psychotherapists to work within the undeniable limitations that are part of ASD?” (Urwin, 2011, p. 247).

While it is beyond the scope of this article to review the full range of psychotherapeutic work with children on the autistic spectrum, the seminal work of Frances Tustin and Anne Alvarez is an important baseline for any psychotherapeutic study of the autistic condition. Tustin’s view of ASD has changed through the years of her work, from her initial view of autism as a defensive regression to a “normal autistic stage” to her conclusion that there is no “early stage of infantile autism” to regress to or halt at. This shift in the understanding of autism is based on work done in developmental psychology, infant observation studies, and the work of researchers, such as Trevarthen (1979), Stern (1985), Piontelli (1992), Alvarez (2004), and others. In Tustin’s later view, autism is a system of protective, but alienating auto-sensual behaviors, developed to deal with an infantile trauma. Children with ASD “live in a predominantly inanimate, sensation-dominated world, one which has limited coherence in virtue of [their] inability to integrate fully their sensory experiences” (Hobson, 2011, p. 233). Inspired by Bion, Tustin conceives bodily sensation as “the archaic element at the foundation of the mind and ultimately of the capacity for thinking” (Mitrani and Mitrani, 2015, xxxii). In her article of 1991, she conceptualizes this condition as “a system of perverse reactions, provoked by a traumatic experience of bodily separateness” (p. 585).

Another cornerstone of the evolution of psychodynamic approaches and interventions is the work of Anne Alvarez, who has questioned the relevance of orthodox psychotherapy in working with individuals with ASD. Alvarez and Reid (1999) define autism as a problem “of intersubjectivity, as a lack of a sense of other persons… an impairment of the normal sense of emotionally based curiosity about, and desire for, interpersonal relationships” (p. 2). Alvarez is cautious about using psychoanalytic terms as “the concepts of transference and counter-transference [which] may seem too advanced: transference may seem to be non-existent, and a counter-transference of frustration or despair in the therapist can lead to indifference. Yet close observation may begin to reveal faint or disordered signs of relatedness which can then be amplified” (Alvarez, 2004, p 96). Alvarez strongly believes that the therapeutic interventions should be attuned to each child’s developmental level and find where the “normal,” non-autistic part of the self is operating, and be “calibrated to the level of emotional communication of which the child is capable – may build” (Alvarez, 2004, p. 93-94). In her own interventions, Alvarez resorts to intersubjective engagement, simple verbal comments, and mainly non-verbal communication. She speaks of being active in “reclaiming” the child back into the world of human feeling and interpersonal exchange (Hobson, 2011). We should note, however, that Alvarez still privileges neuro-typical norms of behaviors which are not necessarily compatible with the perspective of neuro-diversity.

Psychotherapeutic work with autism has thus become self-consciously diverse, taking into account the social and behavioral skills, potential deficits in cognition, and the hypo/hypersensitivities of the clients, and integrating these parameters with developmental psychology and psychoanalytic thinking (Mitrani, 2010). This eclecticism is also evident in understanding that clinicians and other professionals need to “find ways of sharing information in order to establish a ‘communication-enhancing environment’ around the non-communicating child” (Urwin, 2011, p. 245). This recognition leads the common practice of multidisciplinary teamwork of therapists from different professional orientations and areas of specialization, who frequently integrate different treatment modalities to address the complexity of the autistic condition. The process can often be defined as “hybrid intervention” (Thompson, 2013), tailored to the level of functioning of each child, and to his/her particular style and personal needs.

Accepting the need for emotional treatment and the focus on inter-subjectivity still leaves us with the question of how to go about it, as it is clear that exclusively verbal, classic, and orthodox psychotherapeutic interventions are insufficient and perhaps inadequate in and of themselves when working with ASD. The autistic condition affects and sometimes even paralyzes the child’s ability to understand symbolically, his/her thinking becomes excessively concrete, and the abstraction necessary for reflective thinking and imagining is very limited.

In an article on autistic syntax, Dana Amir writes

The autistic child… has no ability for verbal play. His or her use of words is identical to the use of autistic objects. For the autistic child objects are not the substitute for the missing person, but the person him or herself, or part of them, since they yield the sensation the autistic child is yearning for. In the same way, words are not a substitute or a symbol but the thing itself (2013, p. 6).

The range of available psychotherapeutic methods is, accordingly, broad and varied, including use of props, music, dance, art materials, and words as interventions, and psychotherapeutic work aims for a significant impact on the quality of life of the person with ASD and his/her family by working on the social impairment through the therapeutic relationship (Alvarez and Reid, 1999; Wengrower, 2010). Given this diversity of approaches, it is not surprising that prominent psychologists and psychotherapists have emphasized the need for more specifically designated therapeutic techniques (Alvarez, 2004).

Child-centered play therapy features highly on the list of methods and tools for treating emotional aspects of ASD, inasmuch as the concept of “play” integrates well with new understandings in autism research with regard to imitation, ‘theory of mind,’ social skills, and functional and symbolic play skills. The overarching approach of the therapist determines the therapeutic conditions of the play therapy and there are different approaches to facilitating play, or working through play, which therapists use (for details see, Bromfield, 1989; Boucher, 1999; Ruberman, 2002; Josefi and Ryan, 2004; Mastrangelo, 2009).

Expressive art therapies, dance/movement therapy and music therapy are also recognized as effective approaches and tools in the treatment of ASD, as evidenced in the increasing number of studies in the field (Emery, 2004; Martin, 2009; Reschke-Hernández, 2011; Simpson and Keen, 2011; Aithal et al., 2019). Visual art can serve as an alternative means for dialog and communication, and there are many examples of empirical research and case studies describing the art therapist’s work with ASD (Emery, 2004; Lee and Hobson, 2006; Lim and Slaughter, 2008; Stichter et al., 2010; Schweizer et al., 2014, 2019; Van Lith et al., 2017; Malhotra, 2019). The basic assumption is that art serves as an appropriate channel for communication with the child who tends to withdraw from concentrated one-to-one interaction, as it diverts the focus from the personal, intimate relationship by introducing the “art work” as a “third party” (Bragge and Fenner, 2009, p. 18). Like other creative art therapies, and as in the case of play therapy, the approaches differ, including object relations, developmental/behavioral approaches, and psychodynamic approaches. The treatment goals vary accordingly, i.e., from imagination and abstract thinking, through socialization, to sensory regulation, and others (Martin, 2009).

Drama Therapy is another effective approach for working with ASD, and the majority of literature focuses on group therapy work. Research demonstrates that drama therapy can significantly improve empathy, emotional regulation, confidence and self-esteem, creativity and imaginative thinking, and cooperation and social communication skills (Tytherleigh and Karkou, 2010; Greene, 2012; Godfrey and Haythorne, 2013; Haythorne and Seymour, 2016). Tytherleigh and Karkou (2010) argue that drama therapy may offer opportunities to children diagnosed with autism to explore relationships through embodiment play and activities (e.g., sensory-based work, reciprocal cuing and rhythm, and teasing games). Furthermore, they describe projective techniques which may support the development of relationships, group interactions, and role playing and recommend engaging with child’s own worldview and preferred themes and topics for symbolic play. Recent research evaluates the efficacy of drama therapy in teaching social skills to children diagnosed with ASD (D’Amico et al., 2015), and assesses the impact of drama therapy on the early social behavior of children diagnosed with ASD, providing quantifiable evidence that drama therapy has a positive impact on these behaviors (Dooman, 2017).

Music therapy is also a major arena of practice in the treatment of children diagnosed with ASD. Arguably, it is the client group with which music therapy has the highest reputation and has been implemented in the United States and abroad since the 1950s (Dimitriadis and Smeijsters, 2011; Reschke-Hernández, 2011). Individuals diagnosed with autism very often demonstrate significant interest in music, specifically in rhythm, pitch, and harmony, and they often seem to communicate more easily through sound (Dimitriadis and Smeijsters, 2011). While numerous studies explore music therapy with non-verbal clients, current research emphasizes the therapeutic potential of music when working with children diagnosed with ASD who have verbal skills (Thompson and Elefant, 2019; Epstein et al., 2020).

Similarly to the visual arts, music is considered a “non-threatening” medium (Reschke-Hernández, 2011), which is “far from the realms of language” (Dimitriadis and Smeijsters, 2011, p. 115). This mode of work might be just right for an individual who feels more comfortable when the relationship is not based on verbal connecting. Reschke-Hernández (2011) reviews both improvisational techniques and structured techniques used by music therapists. The music therapy described focuses on various aspects of the autistic experience – sensory sensitivities, communication skills (especially expressive communication), social skills, motor and perceptual motor skills, behavior, cognition, and last, but certainly not least, emotional and psychological concerns. Many music therapists turn to the work of Daniel Stern and Trevarthen to highlight the analogy between musical and psychological processes (Dimitriadis and Smeijsters, 2011). Notably, Trevarthen and Malloch claim that the engagement and connection through music can be “a lifeline to human society” (2009, p. 8) for the autistic individual, inasmuch as “we live, think, imagine, and remember in movement” (p. 9).

Dance movement therapists (DMTs) have also been working with clients diagnosed with ASD since the 1960s. This approach relates to the body and its movement both diagnostically and therapeutically (Adler, 1968; Kalish, 1968; Siegel, 1973, 1984; Payne, 1992; Erfer, 1995; Loman, 1995; Parteli, 1995; Torrance, 2003; Tortora, 2006; Wengrower, 2010; Devereaux, 2012, 2017; Samaritter and Payne, 2013, 2017; Martin, 2014; Scharoun et al., 2014; Edwards, 2015; Hildebrandt et al., 2016; Athanasiadou and Karkou, 2017; Aithal et al., 2019; Takahashi et al., 2019). DMT, like other embodied approaches, focuses on “deficits and resources in body movement of individuals with autism spectrum disorder directly as a therapeutic starting point” (Koch et al., 2015, p. 2). DMTs working with clients diagnosed with ASD emphasize the “need to reach these children at their own developmental level that is the primitive sensory-motor level” (Levy, 1988, p. 271), to establish “contact, trust, and rapport,” and to reflect “movements, rhythms, and feelings of the autistic child” (p. 222–223). DMT interventions include various types of mirroring or reflection, contrasting or counter-movement, use of vocalization, sound and rhythm, regulatory movement, non-verbal synchrony (movement synchrony), tuning in and picking up, positioning and movement in space, joining, resonating, matching, echoing, sensory-motor, and perceptual interventions – all broadly aimed at attaining bodily attunement and kinesthetic empathy with the clients (Payne, 1992; Sandel, 1993; Tortora, 2006; Homann, 2010; Wengrower, 2010; Samaritter and Payne, 2013, 2017; Koch et al., 2015; Koehne et al., 2016).

These developments notwithstanding, the particular challenges involved in the emotional treatment of children diagnosed with ASD have yet to be systematically explored. Individual first-person accounts of the lived-experience of therapists in this area mostly feature in specific case studies and clinical vignettes written by psychotherapists. These individual accounts relate to “projective identification,” or “transference and countertransference,” that seem to be specifically related to working with ASD, or to developmental constructs specific to primitive mental states, such as “adhesive identification” (Meltzer, 1975) or “adhesive equation” (Tustin, 1991). Notably, these individual reports are replete with expressions of uncertainty as to the efficacy of the treatment, and sometimes voice a sense of despair or hopelessness. The authors often describe a personal sense of fragmentation, isolation, boredom, and emptiness, psychic numbness, bodily reactions like breathlessness or sleepiness, bodily disintegration, and a sense of their own “autistic experience” (Riley, 1997; Alvarez, 2004; Bergstein, 2009; Fargione, 2013; Durban, 2014; Molinari, 2014; Rhode, 2015). But however candid and detailed, these accounts do not constitute the focal point of these clinical vignettes and case studies described, but are shared as part of the process of therapy and in relation to its progress or lack thereof (with the exception of Bergstein, 2009).

In view of these individually reported challenges, and the concomitant recognition of the complexity of the therapeutic engagement with children diagnosed with ASD, it seems that the responses, interpretations, and coping strategies of therapists working with this client population warrant further systematic and focused research and articulation (Vulcan, 2016). It is all the more important to engage in systematic research on the lived experience of therapists at a time when the prevalence of diagnosed autism is increasing, and the number of professionals who engage with the condition is accordingly on the rise. Understanding the various aspects of the clinician’s experience in coping with this condition may enhance our understanding of the condition itself and shed some light on the efficacy and significance of clinical practices and interventions as well.

## Methodology

The methodological approach was guided by the principles of hermeneutic phenomenology, as formulated in terms of qualitative research by van Manen, aiming to explore “the internal meaning structures of lived experience” (1990, p. 10), and to examine the “insider’s perspective” of therapists working with children diagnosed with ASD, as it is actually lived, rather than theoretically conceptualized. This approach and the concomitant methodology are particularly congenial for psychotherapeutic research, inasmuch as phenomenological inquiry, like psychotherapy, aims at exploring “the internal meaning structures of lived experience” (ibid).

The research questions addressed the perceptions, emotional responses, and interpretations of therapists in relation to the experience of working with children diagnosed with autism. The participants’ responses and reflections are viewed as sources of insights, and the descriptions of their “lived experience” are integrated with their own subjective and reflective process of interpretation (Reid et al., 2005). It is important to note that the description of the experience and its interpretation by the participants are conceived in this framework as a continuum (Finlay, 2009, p. 11), inasmuch as the process of articulating and making explicit a feeling or an experience (that may have only been implicitly or intuitively felt before) often entails a hermeneutic position as well. As the study ultimately aims to be of some pragmatic value to professionals working in this field, “phenomenology of practice” of van Manen (2007) is also highly relevant to the methodological approach underlying this project.

### Pilot Study (Pre-test)

As part of the research, a preliminary study was conducted through lengthy interviews with three novice therapists from different backgrounds (one art therapist, one dance therapist, and one clinical psychologist). The interviews were recorded and later transcribed and analyzed in order to determine the potential value of the project and the preliminary interview schedule (Shkedi, 2003). Following the thematic analysis, the researcher returned to the interviewees in order to obtain feedback on the findings and their interpretation, to meet the required ethical criteria, and to enhance the credibility of the research (Creswell, 1998). Following the results of the pilot study, the research plan was elaborated and consolidated, and the preliminary thematic categories that emerged were taken into consideration in the establishment of the final interview schedule.

### Setting and Participants

Twenty-eight practicing therapists, 24 women, and four men, participated in the study, which was conducted in Israel. The participants were contacted and recruited through purposeful sampling, including only clinicians currently working with children diagnosed with ASD in various clinical and educational settings, such as special education kindergartens and primary and secondary schools. The ages of the children in these settings range from 2- to 16-year olds, but 80% of the therapists interviewed work with children at the younger end of the spectrum, i.e., between the ages of three and seven. No differentiation was made for the purpose of the project between the high- and low-functioning clients, as this heterogeneity reflects the clinical practice *de-facto* in the various treatment settings where the participants work. This decision was warranted by the recent DSM-5 which, as explained above, has merged the previously separate developmental disorders (including Asperger’s syndrome) in recognition of the essential shared features of the autistic condition.

Notably, the majority of institutional treatment programs for ASD in Israel consists of interdisciplinary interventions, inasmuch as they include special-education frameworks, paramedical support (by speech therapists and occupational therapists), and emotional psychotherapy practiced by clinical social workers, psychologists, and creative arts therapists. With regard to emotional psychotherapy, it should be clarified at the outset that in Israel clinical psychologists, creative arts therapists, and clinical social workers are often assigned parallel roles in mental health treatment and educational frameworks for ASD. Within the typical therapeutic constellation in these settings, the therapist meets the client once or twice a week for psychotherapy, and has regular supervision sessions. It was, therefore, deemed important to include the full range of therapists who address the emotional (rather than functional and behavioral) aspects of the treatment. The recruitment was accordingly designed to ensure a broad and diverse range of practice specializations, which are all recognized as modes of emotional therapy. This diversity aimed at illuminating different directions of treatment, and providing detailed and nuanced information about the dynamics of the phenomenon described (information-rich cases). The sample aimed for the widest range of participants with a preference for maximum variation as to the clinical experience (ranging between 1 and 19 years) and the psychotherapeutic specializations of the interviewees. The range of specializations included three clinical social workers, seven clinical psychologists, three visual arts therapists, one drama therapist, 11 dance/movement therapists, and three music therapists Their professional diversity notwithstanding, all of the therapists who participated in this research project share a “psychodynamic understanding of development and psychological functioning… [which is worked] through the interpersonal relationship with the therapist” (Drucker, 2009, p. 36).

In order to ensure confidentiality, neither the names of the individual participants nor their specific professional orientations are given in this article.

### Data Collection

The phenomenological-hermeneutic conceptual framework has thus determined the use of a qualitative research methodology that enables the required in-depth study of subjective experience. In order to examine the construction of perceptions, beliefs, attitudes, feelings, and emotions (Patton, 2002), it is necessary to enter an intimate and nuanced dialog with the participants, to allow their own voice to be heard and to enable their own choice of vocabulary and articulation to constitute the text. This is all the more true for a study of the therapeutic experience, which is by definition elusive, riddled with ambiguities, multi-layered, and fraught with personal, intimate materials which do not lend themselves to quantifiable, statistical studies (Flick, 2002). The ability to tolerate and engage with ambiguity is thus inherent both to the subject matter of this project and to the chosen research approach (Patton, 2002).

The call for participation was distributed *via* professional mailing lists, relevant professional associations, and web sites. In compliance with the ethical codes of research as set by the university ethics committee, all the participants were given full information about the research project, and informed that participation was voluntary and confidential. They all signed a written consent form prior to the interviews, which were audiotaped and later transcribed verbatim in Hebrew. The accuracy of the data was ensured by double-checking the typed transcriptions against the audio recordings. The relevant extracts, chosen as representative of emerging themes, were later translated into English by a qualified translator. The in-depth interviews, spanning between 1 h and a half and 3 h each, were conducted over a period of 24 months, and took place at various places according to the interviewees’ convenience and choice.

In line with the recommended procedures of phenomenological research, the interview schedule was based on the pilot study and addressed topics relevant to the research questions (Rubin and Rubin, 2005): broad data-generating points of reference regarding the participants’ motivation for working with children on the autistic spectrum; the particular characteristics of the clinical experience; physical sensations and emotions triggered in the course of the clinical encounter; examples of particular cases; modes of intervention; modes of processing and interpreting sessions; and the evaluation of therapy outcomes. This interview schedule is not arbitrary, but built from the topics the researcher aims to touch upon, based on prior knowledge, and personal and professional experience of the researcher and on the review of the relevant literature (Rubin and Rubin, 2005). The interview schedule was re-examined and modified after the pilot study. The final interview schedule allowed the interaction to remain flexible enough to allow the interviewees to open up and move on to issues of prime concern or interest to themselves with a minimum amount of interruption or constraint by the interviewer.

In order to reduce researcher bias and enhance the trustworthiness and validity of the data, participant feedback was subsequently sought, and interested interviewees received their transcripts and were offered to relate to them. Participants mostly reported that the categories were representative of their experiences.

### Data Analysis

Interpretative phenomenology involves a conversation that brings together the discourse of the participants, the questions posed by the researcher, the interpretations offered by the participants themselves, and the interpretations offered by the researcher. Following Rubin and Rubin (2005), the data collection, analysis, and interpretation were held simultaneously rather than sequentially, so as to refine the inquiry through a cumulative learning process. The data were analyzed using the procedures of hermeneutic phenomenology as outlined by Benner (1994) and van Manen (1990), namely the “selective highlighting” approaches. Following these methodological guidelines:

1. The therapists’ accounts were read and reread as a whole, in the process of hermeneutically interpreting these texts as inscriptions of lived experience, in order to identify relevant and significant expressions, identify emerging themes, note connections, and group them thematically. As van Manen (1990) notes, the “essential quality of a theme … [is that we] … discover aspects or qualities that make a phenomenon what it is and without which the phenomenon could not be what it is” (p. 107), and a viable theme “seems to touch the core of the notion we are trying to understand” (p. 88). The thematic grouping of the elements which came up in the interviews was based on this characterization of viability.
2. Following this initial reading and re-reading of the interviews as wholes, they were read again with special attention to precise phrases and case examples that related to the experience of working with children diagnosed with ASD. In the course of this focused reading phase, highlighted expressions were identified and clustered into recurrent themes that revolved on significant aspects of the participants’ lived experience. The aim of analysis was to create a comprehensive account of themes which seemed to be of significance. The themes which emerged were based on the original texts of the interviews, rather than on any preexisting theory or theoretical position.
3. On the completion of individual analysis, lists of themes were compared from all interviews, and assembled together as themes within higher order categories.
4. At the point of saturation, which became evident when there was recurrent replication of data concerning the emerging essential themes, marginal themes were dropped (i.e., themes less predominant or non-relevant to the core lived experience), and more prominent themes, found to be fundamental and distinctly related to the experience of therapists working with children diagnosed with ASD, were expanded. The prolonged engagement with the data and the constant reflection and re-examination of the transcripts were undertaken to ensure that the themes distilled in the process were accurately related to the initial material and reflected the original data (van Manen, 1990).

In order to maintain methodological rigor, the transcripts and findings were presented at various stages of the process at qualitative research forums, in a qualitative research group, were offered to participants for “member validation,” and discussed with colleagues, who mostly concurred with the major themes, and added valuable methodological input. Trustworthiness was also established by documenting the process of data analysis in a paper trail (Sandelowski, 1986; van Manen, 1990).

## Findings

### Meeting the Body

Many of the interviewed therapists related to the centrality of bodily experience in the therapeutic encounter, using phrases like “body-to body,” or “being with a body,” a process that “began through the body and gradually seeped into the mind.” One therapist said that the experience involved “an encounter with the primary, the very infrastructure of the body and the soul.” This bodily primacy was specifically emphasized in many of the therapists’ accounts to the difficulties of connecting and communicating through speech. One therapist likened the encounter to a parental relationship, based “on touch, on physical closeness, on cradling, swinging, and jumping, and something that is like, without words to mediate the experience… It is as primary as it can get.” Another therapist referred to the sensory aspect of the bodily connection as “even more primal than the emotional one,” and a similar perception was echoed in various terms by other participants, most notably the analogy to a mother-infant relationship, as explicitly conceptualized by the therapists. For example, one participant said that it is “a benign connection… just like a baby… I am here for you. If you are hungry, I will feed you, if you are tired, I will help you sleep.”

The therapeutic role was described as a kind of parental mediation, “building those islands that may at some points add up to some sort of meaning.” Some participants used the analogy of serving as “a womb” for the child, “wrapping,” “holding,” and “hugging” him or her, as a parent would do. One participant described the difficulties she experienced at end of each therapeutic session as “tearing him [the child] out of a womb,” and another recalled the therapeutic session as “enabling a rebirth.” The parent-infant analogy was carried further by another therapist who spoke of her role as “carrying the child in a baby wrap,” and serving as a “protective envelope” to make up for the autistic experience of the world.

#### Bodily Functions: The Concrete, Material Body

Many of the therapists’ accounts referred to the issue of lower bodily functions, saliva, phlegm, urine, and excrement. Being aware of the potential response of repulsion and disgust at some of the situations encountered in this line of work, the therapists expressed feelings of tolerance and acceptance of these aspects of their work with children diagnosed with ASD, who are often incontinent or unaware of social conventions and boundaries regarding cleanliness and personal hygiene. For example, one therapist said she has “no problem with phlegm, with hands being pushed into various places… it does not bother me.” Another therapist, who said that she did not mind “wiping [the child’s] bottom,” gave a frank example of a child who was very preoccupied with his own body odors, and said that this “did not disgust or shock” her. Connecting this tolerance to a quasi-parental role, another therapist spoke of the child urinating in the therapy room, and said that “at a certain point he becomes my child, and it is not really disgusting for me… [It is] really like my baby.” Some of the therapists regard this aspect of their work as part and parcel of the therapeutic encounter, commenting, as one of them did, “this is what he [the client] brings to therapy, there is no boundary, and he brings it, and it is part of the session.” Another therapist related the child’s urinating in a tub that she had in the room in terms of the child being “born over and over again.”

Unlike the therapists who choose to enable these “regressive aspects” of the child’s behavior and interpret them in a psychodynamic context, others choose to address their interventions to the issue of appropriate socialization and functioning. Speaking of a child’s “runny nose,” one therapist talked about “dripping,” “leaking,” and “clogging,” in the context of social realities, where cleanliness, order, and structure are important. For her, keeping the child clean is all part of the “concrete holding” aimed at making the child aware that “a runny nose is a bother,” and helping him avoid the response of aversion on the part of his caretakers. Another therapist expressed her concern with the issue of “bodily boundary-lines” and the gap between the chronological and the developmental age, evident in the clients’ inability to control lower bodily functions and to understand “where I begin and where I end.”

### Body Oriented Interventions

The centrality of the client’s bodily presence in the therapeutic encounter was also prominent in the therapists’ accounts of their modes of intervention, and some of them explicitly juxtaposed verbal and somatic/kinetic modes of interaction. One therapist said that the latter were “very significant, no less than the verbal messages,” and another participant made a similar distinction between “word” and “voice”: “It is not the words… I think there is more significance to the sensory aspects, voice, smell, movement.” Many of the therapists referred to the relational “positioning of the body in space,” and the issue of physical proximity was recognized by most as significant and essential to the treatment.

Thus, one therapist said that physical closeness is crucial to the dynamics of the process, whereas another spoke of a particular case when she felt a need to “disembody” herself in the therapeutic space, so that the child would approach her. Other therapists noted the “primacy” and the centrality of movement in space as a mode of therapeutic intervention, or as “the starting point” with the difficult cases of autism. References were made to “changing one’s coordinates of position… opening a space or distance” as “the most basic level [of intervention],” which can also serve as a way of “bypassing [spoken] language.” This recognition, shared by several of the therapists, also raised questions like “how close I can come, how to position myself? Should I stay put?,” and the whole issue of positioning oneself in space is, as one participant described it, a process of “trial and error.”

Nearly all the therapists interviewed described their modes of body-based intervention as “joining,” “imitating,” and “mirroring,” the sounds and movements made by their young clients. These terms were used interchangeably by the participants to indicate actions and movements that deliberately resembled those of the child, explained as an intuitive mode of intervention that takes place when it is difficult to “know” what should be done. For example, one therapist said, “I did not know anything, so I imitated him. Whatever he did, I did, too… an autistic kind of imitation [laughs]… That was the channel through which it was easier for him to communicate.” Some therapists related explicitly to bodily and kinetic imitation as an aid which they use to develop empathy with their clients, to “understand [the client’s] experience of being in the world,” and get to the “emotional through the physical experience.” One therapist, recalling her interaction with a particular child, said: “It really helped me to think about her, that is, I used to think about her a lot, but that was one thing, and I felt the need to physically get up and walk like her.” The act of “physical joining” was described as an attempt to listen to “what the movements are saying,” to find “the same language,” to “connect,” or to use the same communication “channel,” as several participants phrased it. The concept of “mirroring” came up again and again, both in the sense of literal-physical replication of the child’s moments, actions, and gestures, and in a figurative sense as related to a sense of selfhood.

#### Touch

Most of the therapists interviewed commented on the inevitability of touch as a therapeutic intervention, and, once again, as a non-verbal “channel of communication.” The main emphasis in the therapists’ accounts was on the impossibility of “keeping things ‘sterile’” when working with ASD, on the necessity of allowing “leaning,” of “holding,” of “giving warmth,” responding to the child’s “need to be hugged.”

While some therapists expressed their acceptance of this aspect of their work, others reported a sense of discomfort at the inevitable physical aspect of the encounter, that touches upon their own issues and inhibitions, and seems to be fraught with a certain “embarrassment” or “confusion” and question of “appropriateness.” The dilemma of allowing touch, even when it is felt to be “invasive” has been translated by one of the participants into a dilemma between taking an “educational” stance (telling the child that it is not appropriate to touch another person’s body) or choosing to respond to that need even at the expense of some personal discomfort.

Several therapists referred to the issue of touch in the context of “parenting”: some of them saw it as “naturally continuous” with caretaking, whereas others reported having an issue with the “crossing of boundary lines” between the parental and the therapeutic role. Notwithstanding the diversity of the therapists’ individual responses to the issue of touch, most of them seemed to agree that “with all the question marks, the physical contact is very fundamental with children with ASD.” Some of the participants recognized their own need for an emotional response, translated into touch: “I need them to come to me for a hug, so that I can go on.”

### “Pathological Optimism” and “wow” Moments

All the therapists interviewed talked about feelings of frustration, helplessness, and even despair encountered in their work, due to the frequent feeling that “nothing happens” and no progress is visible, to a felt lack of reciprocity or consistent feedback. They talked about “attrition” and a recurrent sense of wishing to “give up”; about being anxious and experiencing a sense that, “it may all be meaningless, pointless to treat them.” Many participants talked of the despair generated by the “fluctuations” of their relationships and missing the sense of “connectedness” that is sometimes nonexistent, or at best only intermittent, with these children, “who are with you at one point, and then they are not there.”

The participants described their attempts to counter discouragement by a conscious effort to “to believe and to hope,” even if “there are moments when it is difficult to keep on believing,” but some of them voiced their concern that this willed hope is merely a kind of necessary “fantasy,” “self-delusion,” or “wishful thinking” on their part. The need to believe was explicitly referenced by one participant as a kind of “denial,” and another spoke of the “need to be ‘stupid’ and hold on to some sort of pathological optimism” in order to continue with this “unrewarding, hard work” that can “burn out one’s batteries.” The possibility of hope was presented in many of the accounts as based on the ability to see the most minute and “almost invisible” signs of progress, when moments of felt accomplishment are brief and sometimes difficult to articulate or fleeting. The therapists talked of moving in “tiny-weeny steps,” of needing “a magnifying glass” in order to see these minute indications of progress, or “the god of small things.”

Notably, the therapists’ accounts of rare moments of connection with their clients, the “moments of magic,” as one therapist called them, were often related to unmediated, non-verbal bodily interactions, characterized by a physical combination of sound, rhythm, posture, or gesture, and associated with primal, bodily attributes. One therapist said.

Suddenly, there was a look, we listened to “something… something rhythmic, a sort of accompaniment, and I was on the floor and on the rug and then something happened, ah, some connection was made, it was like a moment of Wow, this is happening, it is like something is working.”

Other therapists also recalled vignettes involving their clients’ responses at such somatic or kinetic exchanges, and moments of connectedness elicited non-verbally, “from gut to gut” through music, “eye contact and then touch,” a smile or a gesture of “reaching out,” or “giving me a first hug.” These responses may be minute and almost invisible, such as “nuances of tonus and muscle, perhaps the way the finger moves… A heartbeat or breath or look,” but they are construed as signs of recognition and reciprocity. One therapist said it was enough for her to have the child put his hand in hers when she arrived; a gesture of recognition which, she said “[was] already, like ‘Wow’ for me.” For some of the therapists interviewed, the moment when they, too, let go of the verbal aspect of communication was the point when they felt they had attained insights, which are often non-theoretical, but nonetheless real and significant. As one therapist put it, letting go of the words is not easy, but “it can be done, and it is magical.”

## Discussion

The aim of this study was to explore the experience of working with children diagnosed with ASD, as it is perceived and processed by therapists from various professional orientations. The general approach of participates is compatible with the traditional diagnostic view of ASD as a condition, and none of them seem to adopt perspective of neurodiversity. The study may therefore be framed as suggested by a reviewer as “an examination of the intersubjective experience of neuro-typical practitioners working with neuro-diverse populations, the outcomes of which were the discoveries that a focus on embodiment and embodied-kinetic process may be an effective strategy for neuro-typical practitioners to engage in therapeutic interventions with neuro-diverse individuals.”

The most notable overarching theme was the primary nature of the encounter and the strong sense of the child’s bodily presence in the therapeutic session. Significantly, the bodily focus was not confined to the accounts of therapists who can be seen as having a somatic orientation at the outset (e.g., music or dance/movement therapists). It came up very forcefully in all of the therapists’ accounts, regardless of their initial professional orientation and type of practice. The role of body and movement in the therapeutic encounter was perceived by most interviewees as compensating for the deficiency in intersubjective verbal communication, an alternative, intuitive, and implicit mode of connectedness.

A relevant conceptual matrix for understanding this commonality may be found in “interaction theory” of Gallagher (2004) of autism. Building on constructs of Trevarthen (1979) of “primary” and “secondary intersubjectivity,” Gallagher (2004, p. 205) suggests that “pretheoretical (nonconceptual) sensory-motor capabilities for understanding others already exist in very young children” and that “this sense is implicit, at least in a primitive way, in the behavior of the newborn.” In the case of autism, according to Gallagher, it is precisely these capabilities which are impaired (for further elaboration on the phenomenologically informed clinical approach to autism, see Nilsson et al., 2019). It appears then, that the therapists interviewed have intuitively acted upon this conception of autism in search of a point of primary connectedness with their clients. Rather than relate to “Theory of Mind” (Baron-Cohen et al., 1985), the therapists related to the bodily encounter with their clients as the springboard for the development of primary and secondary intersubjectivity.

As noted in the introduction, individual accounts of working with ASD often relate to the challenges posed by the “quicksand of hopelessness, lifelessness, and meaninglessness” (Riley, 1997, p. 63). Indeed, the participants expressed frustration or even despair at the lack of connectedness, and the difficulty of establishing an inter-subjective relationship with their clients. However, what has emerged from the interviews can also be construed as an antidote to despair, based mostly on “magical moments” of bodily being and bodily relatedness. Notably, these “WOW” moments, the moments of connection and felt progress, were reportedly triggered by non-verbal and unmediated somatic interventions reinforcing that bodily “implicit right brain-to-right brain intersubjective transactions lie at the core of the therapeutic relationship” (Schore and Schore, 2008, p. 14). The therapists chose to speak of “bodily knowledge” as an enabling tool or a pathway to a better understanding of their often-nonverbal clients. This can be understood as part of the “clinical reasoning” described by Gallagher and Payne (2015). Whereas “reasoning” usually refers to intellectual, cognitive, and higher level processing (reflecting, selecting means, evaluating outcomes, etc.), in the therapists’ descriptions it acquired a different slant, inasmuch as they spoke of their understanding, intervention, and feeling of “what actually works” as part of “intuitive knowledge” gained in an “intersubjective context” (p. 69).

While these interventions were described by the participants as intuitive moves when one is “at a loss for words,” they can arguably be validated by theoretical constructs drawn from the field of developmental psychology, dance/movement therapy, and the phenomenological approach to autism. One concept of particular relevance in this context is Stern (1997) notion of “formulating the unformulated”: verbalizing an action, without misrepresenting or losing its essence, expressing what is “not represented in words at all, but in the semiotics of practice” (p. 18). Other relevant constructs are those that refer to the role of non-verbal communication and sensory motor interactions – “somatic countertransference” “affect attunement,” “kinaesthetic empathy,” “resonance,” “mirroring,” “somatic intersubjective dialog,” and “embodied attentiveness” (Kestenberg, 1975; Trevarthen, 1979, 2004; Stern, 1985; Rhode, 2005; Bloom, 2006; Meekums, 2007; Vulcan, 2009, 2013; Wengrower, 2010; La Barre, 2011; Devereaux, 2012; Koch et al., 2015). Notably, all of these constructs are relevant both to parent-infant relations and to therapeutic interventions with children diagnosed with ASD.

The concept of “psycho-sensory reverie” developed by Lombardi is also significant in this context. Lombardi’s focus on the primitive experience of the body goes back to Freud and Bion in search of the roots of the body’s involvement in the psychoanalytic interaction. His focus on music notwithstanding, Lombardi (2008) notes that the psycho-sensory reverie can be visual, olfactory, kinaesthetic, etc., no less than auditory or musical. This use of psycho-sensory reverie as a tool for processing the somatic raw data brought by the ASD client into the session is probably what accounts for a sense of guilt or frustration on the part of the therapists, who spoke of their sense that “nothing happens” during the session. One may suggest, then, that if these constructs were part of the conceptual framework for a bodily oriented training, therapists would have felt less guilty and more self-confident about their perceptions of what actually happens in the therapeutic encounter and of the clinical significance of psycho-sensory reverie. It appears, then, that the intuitive sensory, somatic, and kinetic interventions described by the participants may offer some relevant insights into the process of supporting the development of both inter-subjective relatedness and (cognitive and emotional) mentalization in children with ASD.

The recognition of the centrality of the body within the therapeutic encounter suggests that the very foundation of an inter-subjective relationship – the ability to recognize self and other, to empathize, to imagine the inner world of the other – may indeed be generated by an embodied relationship. As noted by Koch et al. (2015) “the mind is not hidden but directly expressed in other persons’ embodied actions’ from the earliest days of infancy” (p. 2). While this is generally true of the parent-infant relationship, where the dyadic relationship is based on the ability to understand each other’s bodily affect-loaded behavior in the early stages of attachment, it is arguably also directly relevant to the pathology of Autism.

One may conclude, then, that the concrete bodily interaction between the therapist and child is relevant to the “muscles of the psyche,” to borrow the expression used by one of the participants. The findings suggest that it is not enough for the therapist to be attuned to the text of the body and relate to its potential meaning. For those “Wow moments” to occur, the therapist should relate to the concrete body as the text itself and initiate his/her interventions on the somatic register. We may thus add another dimension to Tustin (1992) use of body-oriented “active responses” on the part of the therapist: the body and its manifestations are not only objects for verbal interpretation, but constitute the text itself for both participants in the interaction.

Indeed, the focus on the somatic aspects of primitive mental states and the bodily precursors to psychic structures is not in itself a new contribution (e.g., Bick, 1968; Meltzer, 1975; Tustin, 1981, 1991; Ogden, 1989; Haag et al., 2005; Rhode, 2005; Mitrani, 2010; Amir, 2013). However, the potential new contribution of the findings presented here lies in additional focus on somatic motility, inasmuch as it is not only the body that needs to be observed and interpreted, but also the actual *moving body* which is fundamental in the formation of subjectivity. It is, then, arguable that motility can play a major role in therapeutic interaction and intervention. To frame this contribution, we may turn to the early work of Khan (1974), which relates to the client’s active “kinetic being.” Khan speaks of having learned to “accept that often self-experience in the analytic situation can have no means of symbolic and/or concrete actualization if motility is rigidly tabooed. Self-experience is intimately related to body-ego” (p. 297). This focus on motility from a developmental perspective is at the core of the work of psychotherapists like La Barre, who has coined the term, the “kinetic text” (2011) to account for this dimension of the psyche. Needless to say, the recognition of the interconnectedness of motility and psyche is the very foundation of dance/movement therapy from its very inception (Bloom, 2006).

A lesser known but significant theoretical contribution to the concept of motility as a precursor of a sense of selfhood has been offered by the Israeli movement theorist, Yona Shahar-Levy, whose understanding of the connection between psyche and movement goes well beyond the psyche-soma relationship. Shahar-Levy (2001, p. 381) focuses on the role of motility and articulates the recognition that “for the growing infant, movements are never dissociated from emotive–relational states, and emotions are never divorced from motility. Emotive contours always contain motility contours and vice versa.” Early primary motility thus has a major role in the later development of the psyche and the formation of the sense of self, and Shahar-Levy makes a convincing case for the significance of “emotive motility,” as a potential point of entry into the psyche and for “correction” and “healing” (p. 392). The findings of this research confirm that the emotional connection between the child and the therapist is made on a pre-cognitive, primal and bodily level, and that motility and “shared movement” may become the means for creating a “new way of being” with these children (Samaritter and Payne, 2013, p. 148).

Reading the participants’ accounts of what happens *de facto* in the therapeutic encounter, regardless of their diverse professional orientations; we may conclude that the training and education of therapists who prepare themselves for work with children diagnosed on the autistic spectrum should include a significant emphasis on the bodily self, movement, and somatic awareness. As Sletvold (2012) suggests, “by incorporating embodied practices from bodywork and the performing arts, analytic training could be enriched and promote the development of countertransference awareness for body sensations and movement” (p. 410). These clinical implications may be further reinforced by the work of Trevarthen (2004), who points to the need for a “model of non-verbal communication based upon acceptance of intrinsic affective states” (p. 11), inasmuch as “non-verbal communication, including improvised music and movement and touch therapies, can help a variety of disorders affecting a young child’s relating, including autism” (p. 10). Training the “muscles of the psyche” through embodied practices should, then, be integrated into curricula development for therapists who prepare themselves for work with clients diagnosed with ASD. Such embodied practices may enrich the clinician’s tool kit by enabling her/him to develop an awareness of body sensations and movement, recognize embodied resonance, become conscious of somatic transference and counter transference, and foster kinesthetic empathy in understanding their client’s experience of the world “from within.”

## Limitations

The findings of this qualitative study have emerged from an exploration of a small number of detailed in-depth accounts given by therapists of different professional backgrounds, all of whom practice within a psycho-dynamic rather than a cognitive-behavioral perspective. The professional orientation of the participants is undoubtedly relevant to their lived-experience, as reported in the findings. If we wish to get a broader and more general understanding of the issues involved, it may be worthwhile to supplement the findings of this project by further research into the lived-experience of psychotherapists with cognitive or behavioral professional orientations. Another perspective which may be valuable to explore is that of cultural neuro-diversity, which did not seem to resonate in the interviews with the clinicians in this study. Future research should involve clinicians, who identify themselves as working, first and foremost, within the neuro-diversity paradigm to understand if their experience is fundamentally different from that of the therapists described in this research, who did not embrace this perspective.

A further potential limitation is related to the sample. Although the selection of participants aimed at maximum diversity in terms of their professional areas of specialization, 11 out of the 28 respondents were DMTs. This may well be taken as a potential limitation, due to the somatic orientation that DMTs bring with them to their work at the outset, and which may have affected their responses as an “overvalued idea.” I would suggest, however, that the similarity of responses across the various disciplines – DMTs, clinical social workers, and child psychologists alike – points to the primacy of the encounter and the place of the body in the intersubjective process as perception that goes well beyond the specific training and orientation of the participants. Another point to bear in mind in this context is the focus on the lived experience of the therapist. This perspective was explored as a potential avenue toward a better understanding of the autistic condition itself, but one should exercise caution in making these inferences automatically. Evidence based research on autism and first person accounts of high-functioning people with autism point to some differences in cognitive and psychic structures (Baron-Cohen, 2010; Mitrani, 2010), and it is important to avoid sweeping conclusions, so as not to impose the experience and feelings of the participating therapists on those of their clients, as many first-person accounts hold invaluable data of the experience (e.g., Grandin, 1995; Ashby and Causton-Theoharis, 2009; Fleischmann, 2012; Gratton, 2019; Tesfaye et al., 2019).

## Conclusion

The essential themes that emerged from this qualitative study related to the centrality of the body and of bodily motility in the therapeutic encounter with children diagnosed with ASD, to the difficulties of intersubjective relations, and to the potential effectiveness of somatic and kinetic interventions. The specific focus of this study indicates that some of the emotional and relational challenges involved in the autistic condition may be addressed through an embodied perspective. The implications of this study are potentially relevant to the training and support of therapists working with ASD, but further research is warranted in order to enhance the current theoretical understanding of the centrality of the body and the clinician’s role in this type of therapeutic encounter.

A good starting point for the development of relevant somatic and kinetic modes of therapeutic work may be found in the practices of embodied psychotherapies. These approaches, while playing a major and significant role in actual clinical practice with children on the autistic spectrum, are still often marginalized in terms of professional and public recognition, and seen as merely “supplementary” modes of treatment (Vulcan, 2013). The findings of this research project suggest that these embodied approaches should be given adequate recognition and sufficient scope for developing their particular tools, and that integrating different languages of psychotherapeutic work can assist in understanding the emotional inner states of clients on the autistic spectrum, and perhaps of other clients as well.

## Data Availability Statement

The raw data supporting the conclusions of this article will be made available by the authors, without undue reservation, to any qualified researcher.

## Ethics Statement

Ethical review and approval was not required for the study on human participants in accordance with the local legislation and institutional requirements. The patients/participants provided their written informed consent to participate in this study.

## Author Contributions

MV designed the study and carried out the data collection and analysis. The manuscript was written by MV.

### Conflict of Interest

The author declares that the research was conducted in the absence of any commercial or financial relationships that could be construed as a potential conflict of interest.
