# Supplementary material for: The “Muscles of the Psyche”: From Body Literacy to Emotional Literacy
Source: Front Psychol. 2021 Jan 20;11:548964. doi: 10.3389/fpsyg.2020.548964 (PMC7854468; doi:10.3389/fpsyg.2020.548964)
Supplement: Supplementary file 1 [file Data_Sheet_1.pdf]

Table 1 Respondents' demographic information.

Pseudonym	**Sex and age**	**Profession**	**General years of experience**	**Years of experience working with ASD**	**Other roles in this line of work**	**Experience with other client groups**
Saar	M, 31	Psychologist	4	2	Group therapist, parental guidance	Yes
Lihi	F, 34	Art therapist	2	2	None	Yes
Saggit	F, 48	Dance therapist	17	17	Parental guidance, staff supervision	Yes
Alma	F, 32	Dance therapist	2	2	Kindergarten teacher	No
Anna	F, 55	Art therapist	10	10	Case manager, supervisor, parental guidance	Yes
Hillit	F, 48	Music therapist	16	11	Case manager, supervisor, parental guidance	Yes
Meirav	F, 36	Music therapist	2	2	None	No
Limor	F, 50	Art therapist	19	19	None	Yes
Gilly	F, 30	Dance therapist	3	3	None	No
Sivan	F, 43	Dance therapist	11	11	Supervisor	Yes
Michal		Music and dance therapist	16	15	Parental guidance	Yes
Liora	F, 35	Drama therapist	4	1	None	Yes
Avigail	F, 33	Psychologist	1.5	1.5	None	No
Lilach	F, 37	Psychologist	7	1	Kindergarten teacher, case manager, supervisor	Yes
Danny	M, 37	Psychologist	10	2	Kindergarten psychologist, case manager, parental guidance	Yes
Tamar	F, 35	Clinical social worker	10	2.5	Yes (unspecific)	Yes
Debbi	F, 55	Psychologist	25	9	Head psychologist	Yes
Tali	F, 38	Psychologist	5	1	Case manager, parental guidance, staff supervisor	Yes
Taniya	F, 39	Clinical social worker	9	2	Case manager, parental guidance, staff supervisor	Yes
Guy	M, 40	Music therapist	5	3	Case manager, parental guidance,	Yes
Avishag	F, 42	Dance therapist	18	11	Kindergarten teacher, case manager, supervisor	Yes
Gilad	M, 38	Music therapist and clinical social worker	8	8	Supervisor	Yes
Ruti	F, 35	Dance therapist	7	7	Yes (unspecific)	Yes
Orit	F, 39	Dance therapist	9	7	Case manager, supervisor	Yes
Ailona	F, 45	Art therapist	14	13	Case manager, parental guidance, staff supervisor , group therapist	Yes
Shira	F, 31	Psychologist	3	2	None	Yes
Sandra	F, 28	Dance therapist	3	1	Staff guidance	No
Roni	F, 42	Dance therapist	8	4	Supervisor	Yes
